# Understanding People With Chronic Pain Who Use a Cognitive Behavioral Therapy–Based Artificial Intelligence Mental Health App (Wysa): Mixed Methods Retrospective Observational Study

**DOI:** 10.2196/35671

**Published:** 2022-04-27

**Authors:** Saha Meheli, Chaitali Sinha, Madhura Kadaba

**Affiliations:** 1 Department of Clinical Psychology National Institute of Mental Health and Neurosciences Bangalore India; 2 Wysa Inc Boston, MA United States

**Keywords:** chronic pain, digital mental health, mobile health, mHealth, pain management, artificial intelligence, cognitive behavioral therapy, conversational agent, software agent, pain conditions, depression, anxiety

## Abstract

**Background:**

Digital health interventions can bridge barriers in access to treatment among individuals with chronic pain.

**Objective:**

This study aimed to evaluate the perceived needs, engagement, and effectiveness of the mental health app Wysa with regard to mental health outcomes among real-world users who reported chronic pain and engaged with the app for support.

**Methods:**

Real-world data from users (N=2194) who reported chronic pain and associated health conditions in their conversations with the mental health app were examined using a mixed methods retrospective observational study. An inductive thematic analysis was used to analyze the conversational data of users with chronic pain to assess perceived needs, along with comparative macro-analyses of conversational flows to capture engagement within the app. Additionally, the scores from a subset of users who completed a set of pre-post assessment questionnaires, namely Patient Health Questionnaire-9 (PHQ-9) (n=69) and Generalized Anxiety Disorder Assessment-7 (GAD-7) (n=57), were examined to evaluate the effectiveness of Wysa in providing support for mental health concerns among those managing chronic pain.

**Results:**

The themes emerging from the conversations of users with chronic pain included *health concerns*, *socioeconomic concerns*, and *pain management concerns*. Findings from the quantitative analysis indicated that users with chronic pain showed significantly greater app engagement (*P*<.001) than users without chronic pain, with a large effect size (Vargha and Delaney *A*=0.76-0.80). Furthermore, users with pre-post assessments during the study period were found to have significant improvements in group means for both PHQ-9 and GAD-7 symptom scores, with a medium effect size (Cohen *d*=0.60-0.61).

**Conclusions:**

The findings indicate that users look for tools that can help them address their concerns related to mental health, pain management, and sleep issues. The study findings also indicate the breadth of the needs of users with chronic pain and the lack of support structures, and suggest that Wysa can provide effective support to bridge the gap.

## Introduction

### Background

Chronic pain is a debilitating condition that affects over 2 billion people worldwide, with the global weighted mean prevalence estimated to be 30.3% [1,2]. The sequelae of chronic pain are spread across the different aspects of an individual’s life, such as employment, sleep, physical and mental health, and social relationships [3].

The relationship between chronic pain and mental health comorbidities is well documented, with the prevalence of concurrent major depression or anxiety diagnoses ranging between 35% and 50% of the global population, and the presence of pain often doubling the risk of having a mental health diagnosis [4,5]. Mental health concerns can compound the difficulties in gaining access to affordable and accessible treatment for chronic pain. The absence of referral pathways, high costs, and stigma increase barriers to access for treatment and management [6-8]. Increasingly, digital health care apps have been addressing these challenges with cost-effective and accessible solutions, with some evidence to indicate feasibility and efficacy [9,10]; however, adoption, acceptability, and quality still remain challenges for this space [11]. Currently, there is limited user-led research that captures the perspectives and perceived needs (based on the person’s own judgement) of individuals with chronic pain.

This study examined the experiences of users with self-reported chronic pain who were using Wysa, which is an anonymous artificial intelligence (AI) conversational agent app for digital mental health. A previous study showed that the use of Wysa was associated with a significant reduction in depressive symptoms in a high-engagement user group compared with a low-engagement user group [12]. The Wysa app uses a free-text conversational interface to listen and respond to the user and responds to the user’s distress by recommending evidence-based elements from cognitive behavioral therapy (CBT), behavioral reinforcement, and mindfulness, among others. The self-help practices and conversation-based tools and techniques provide support for challenges, including anxiety, sleep, low energy, motivation, loss, and pain.

### Our Study

The purpose of this study was to understand the perceived needs of individuals with chronic pain and their mental health concerns. The study aimed to understand the ways in which individuals find digital health support meaningful, and the potential areas in which it could be developed in order to offer more effective and accessible support for individuals with chronic pain.

This study had the following 3 objectives: (1) to evaluate the perceived needs of users with chronic pain conditions; (2) to evaluate the app engagement and disengagement patterns of users with chronic pain (in terms of the most used tools, most frequented conversational flows, and intensity of engagement and disengagement); and (3) to evaluate the efficacy of the Wysa app and its interventions for improving mental health among a subsample of users who have completed 2 questionnaire assessments (ie, preintervention and postintervention) using the validated Patient Health Questionnaire-9 (PHQ-9) [13] and Generalized Anxiety Disorder Assessment-7 (GAD-7) [14].

## Methods

### Ethical Considerations

The Wysa app is publicly available as an app on the Android and iOS app stores. It has been designed to prioritize safety, privacy, and security by design. There is no user registration required, and no personally identifiable information is asked at any time during app use. As the study involved analyzing real-world data from an anonymous nonclinical population, it was exempt from registration in a public trial registry (according to OHRP guidelines [15]). The users voluntarily downloaded the app after having consented to the app’s Terms of Service and Privacy Policy. For ethical and privacy reasons, the authors did not have access to all the user messages. Only minimal and limited conversational data extracted based on keywords were used for this research, and no longitudinal data were used. The study data set was deidentified using one-way cryptographic functions. User data were adequately secured according to the organization’s privacy, security, and safety policies. The study participants were informed about how they can exercise their rights to restrict processing of their data for research purposes.

### Study Design

During the study period from October 2020 to October 2021, a total of 2194 users were identified to have reported chronic pain based on selected keywords in ongoing conversations with the conversational agent. We implemented a retrospective observational study with a mixed methods approach, given the objectives of the study and the nature of the data being analyzed. In a retrospective observational study, the sample is defined later and the data are already available [16]. This methodology has been used in multiple health care studies, such as those involving depression and anxiety [17-19], hypertension [20], postpartum mental health concerns [21], COVID-19–related concerns [22], and diabetes [23].

### Outcome Measures and Data Types

The following measures and data types were used: (1) textual snippets from users, (2) tool usage data, (3) usage data indicating the interventions used with the bot, and (4) self-reported PHQ-9 and GAD-7 data.

### Procedure

#### Data Extraction

To extract the data, the first step was to optimize the keywords. For this, the classification of chronic pain by the International Association for the Study of Pain was used as a guide [24] to derive keywords. Additionally, other relevant keywords were derived from literature on pain and the clinical experiences of the researchers in this study. As such, the keywords also included general terms used to describe pain or to describe pain-related experiences, such as “pain,” “painkiller,” “manage pain,” “nerve block,” etc.

User messages that had at least one or more of the keywords were extracted, which resulted in 83,000 messages. The criteria for data extraction and filtration are listed in Textbox 1. These were further filtered by the research team, who read through each of the messages to exclude any pain that did not relate to chronic pain conditions (such as emotional pain or menstrual pain) or pain that the users did not experience themselves. Ambiguous messages that did not clarify the nature of the pain were also excluded. For example, the word “pain” often created false positives or ambiguity, as users could use this word in a context that would not necessarily be related to physical pain or chronic pain (eg, “it is so painful”). These types of messages were excluded from the final set. The stringent exclusion resulted in a final set of 3300 relevant messages from 2194 unique users. Among these, the distribution of the number of mentions of different diagnoses is listed in Table 1.

**Criteria and keywords for data extraction.**

**Selection criteria (inclusion and exclusion) for data extraction**

1. The keywords used were about “chronic pain” or related concerns to capture all mentions of associated conditions, such as “disability,” “loss of limb,” “manage pain,” “injury,” “musculoskeletal,” “neuralgia,” “back pain,” “phantom pain,” “multiple sclerosis,” “osteoarthritis,” “cancer pain,” “sciatica,” “fibromyalgia,” “spinal cord,” “arthritis,” “spondylitis,” “cervical,” “rheumatoid,” “endometriosis,” “surgery,” “ankylosing,” “inflammation,” “inflammatory,” “crohn,” “sclerosis,” “gout,” “nerve block,” “analgesic,” “painkillers,” “pelvic pain,” “cyst,” “migraine,” and “pcos.”
2. The report of pain should be first-person, that is, the person is talking about their own pain. Any mention of others’ health conditions was excluded.
3. There was clarity about the mentioned pain being chronic and physical in nature. Ambiguous messages that did not meet this criterion were excluded.
4. Any message that qualified only as emotional or psychosomatic pain was excluded.

**Table 1:** The number of mentions of different conditions of chronic pain.

Condition	Number of mentions^a^
Migraine	551
Back pain	501
Chronic pain	265
Surgery	212
Fibromyalgia	119
Injury	99
Disability	92
Neck pain	56
Arthritis	30
Crohn	22
Sciatica	19
Inflammation	18
Cervical	9
Rheumatoid	9
Sclerosis	9
Endometriosis	8
Spondylitis	5
Cyst/polycystic ovary syndrome	5
Multiple sclerosis	4
Nerve block	2
Phantom pain	1

^a^This is the number of actual mentions of the condition and not the number of users having the condition. Some users reported multiple conditions, and these categories are not mutually exclusive.

### Analyses

#### Objective 1: Perceived Needs of Users With Chronic Pain

Inductive thematic analysis [25] was used to gather information on perceived needs and experiences related to chronic pain. The author SM got familiarized with the data by reading the messages multiple times. This was followed by the generation of preliminary codes, which were then grouped into potential subthemes and themes. The data were then verified for relevance to the respective themes at each level, and the initial set of themes was verified in relation to the coded extracts. The themes were selected and finalized based on their relevance to the objectives of the study and their salience in capturing perceived needs. Though the analysis was led by SM, all the authors met at regular intervals to ensure that the themes were internally consistent and unique, and answered the research question. The themes and subthemes were then reviewed and finalized.

#### Objective 2: App Engagement and Disengagement of Users With Chronic Pain

The engagement of users was evaluated at the following 3 different levels: (1) the frequency of usage within Wysa, (2) the intensity of user engagement, and (3) the points of disengagement. Engagement refers to the number of initiated and completed interactions within the app, while the points of disengagement are instances where users stop communicating or engaging further with the conversational agent.

The first level of analysis was the frequency count of the tools (interventions) most utilized by the users with chronic pain (N=2194). To capture the most recurring conversation flows of the users with chronic pain, a network analysis [26] was conducted with cleaned sequential pairs of conversational path units and their frequency of occurrence as weights. The path units were then grouped into higher units based on app elements, and the networks were visualized in Gephi 0.9.2 [27] as a directed weighted network. The directionality of the conversational pathways was then used to identify the tools most used within Wysa, to further validate the findings of the first-level analysis. The visualization of the network analysis was done in Gephi with the Yifan Hu layout, and the appearance of nodes and edges was based on “weighted in-degree,” that is, incoming connections as calculated within the software.

At the second level, to examine the intensity of engagement, the extracted data were analyzed for instances of engagement and disengagement with the bot. Using Python 3.6 code, the first-level engagement of a user was calculated by using the number of conversational pathways per user. The intensity of engagement and disengagement of users with chronic pain was then tested in comparison to users without chronic pain (a randomized sample from the larger user base that had not reported any chronic pain). The data for both samples did not fit the criteria for normality, indicating the need for nonparametric assessments. The data were further tested using R 4.1, and the difference in engagement was assessed using the Mann-Whitney *U* test between users with chronic pain and those without chronic pain.

To evaluate the disengagement patterns, the ends of conversational flows, which represented the locations where users would stop engaging with the app, were identified for both groups. The top 20 conversational ends were located from path data and compared between the groups (users with chronic pain, n=2194; users without chronic pain, n=1880) for salient differences.

#### Objective 3: Efficacy in Improving the Mental Health of Users With Chronic Pain

A subset of the sample was used to examine the efficacy of the app for improving the mental health symptoms of users with chronic pain. This subset was restricted to users who completed at least two self-reported PHQ-9 (n=69) and GAD-7 (n=57) assessments during the study period. The first assessment during the study period was regarded as the baseline, and the last assessment was regarded as the postintervention assessment. This subsample was further restricted to users with a score greater than 5 on either the PHQ-9 or GAD-7 (mild distress or greater) at baseline. A paired *t* test was performed between the first and last assessments of the study period for this subset of users with chronic pain.

## Results

### Objective 1: Perceived Needs of Users With Chronic Pain

**Figure 1:**
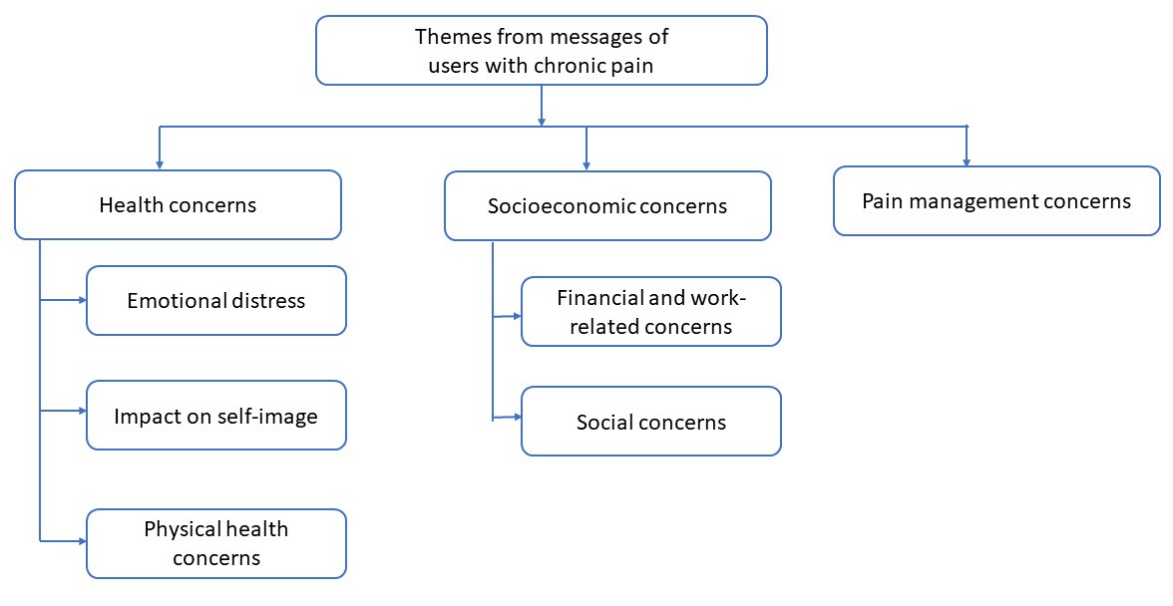
Themes of messages from users with chronic pain (N=2194).

The thematic analysis yielded themes representative of the perceived needs and concerns of users with chronic pain. The themes (given in Figure 1) are *health concerns*, *socioeconomic concerns*, and *pain management concerns*.

#### Health Concerns

This theme indicates the perceived needs, concerns, and experiences of users with regard to their mental and physical health. It includes the subthemes of *emotional distress*, *impact on self-image*, and *impact on physical health.*

##### Emotional Distress

The users with chronic pain would often write to Wysa about strong emotional difficulties, such as anxiety, feeling low or depressed, panic attacks, frustration, and irritability, among others. Many were often concerned with how chronic pain tended to increase their stress and affect their mental health. Some found themselves “flooded with suicidal thoughts and negative emotions.” This concern for the effect of chronic pain on one’s mental health was clearly demonstrated by a user who reported “it’s taking a big toll on my mental health.” The relationship between emotional distress and pain would often be cyclical or bidirectional, with some reporting how their stress levels often aggravated the pain, for example, “I have lupus, fibromyalgia, and asthma, and they are triggered by my stress levels.”

##### Impact on Self-Image

Chronic pain users often reported low self-confidence, with one user stating, “I am still young and having such a condition makes me feel inferior to other teens my age. I feel weak and useless” and others stating that they had “lower self-worth,” with the limitations impacting them deeply. A user said, “I have fibromyalgia, and it makes me feel like a failure at times.” Multiple users also reported having body image concerns, with some feeling “ugly” or feeling ashamed of how they appeared.

##### Impact on Physical Health

Apart from the significant pain experienced, this group of users also reported a variety of issues related to their physical health. Many indicated disturbed sleep (“I can never sleep well”) and constant physical discomfort (“which means I can’t sit or stand or sleep properly”). They also worried about the possibility of their pain worsening and further physical distress. Some were “scared” of their upcoming surgeries. An often-reported issue was the limitations felt by users around physical activities and the resulting distress.

#### Socioeconomic Concerns

The experiences that users with chronic pain shared with Wysa included their experiences of their social relationships and their experiences related to work and finances. The subthemes under this theme are *financial and work-related concerns* and *social concerns.*

##### Financial and Work-Related Concerns

Users with chronic pain often reported several financial and work-related concerns. While some reported that they were unable to perform to their full potential due to chronic pain, others stated that chronic pain was “increasing work stress” and the possibility of “burnout.” The pain would be so debilitating at times that they would often find themselves unemployed, as highlighted by another user who stated, “made it hard for me to find work.” This work-related concern was also demonstrated in a response from a user.

I had to leave work early because of back pain. I feel inadequate for not being able to work with this body. It's frustrating and I feel like a burden.

This concern was further compounded by worries about the future, for example, a user wrote, “make(s) me worry if I will be able to survive in a capitalist system and if I will be able to sustain myself,” and financial worries about therapy and medical bills. Another user made the below statement.

Nothing I have tried for fibromyalgia has helped. I can't afford therapy. I can't afford food. I'm scared about everything all the time.

##### Social Concerns

Users with chronic pain often wrote to Wysa about how the pain limited their social interactions. This was clearly illustrated by a user who wrote, “couldn’t go out with my boyfriend to meet some friends” to portray how chronic pain could restrict someone’s mobility, preventing them from going out and socializing. Some users also reported how the pain and related health issues made them withdraw socially and reported feeling “lonely*.*” They also felt isolated in their pain and reported the feelings of not being understood, not receiving help in seeking professional help, and not being supported.

Well, I am in a relationship but seriously considering a split; he doesn’t help, he’s more of a hindrance. He argues with me when he's asked to help. I have chronic pain. He picks at the triggers as if it’s a game.

Some also talked about the gratitude they felt for being supported by their family members (“Even though I had a terrible migraine today, my husband just made me feel like I was his whole world. I felt so loved”), which further highlighted the need for social support in individuals with chronic pain. This wish for validation and support was expressed by a user who wrote to Wysa as follows:

I am diagnosed with a bunch of things like compressed disk and pain in my back and I also have arthritis in both of my knees … and a couple more things like neck problems. So, I just want everyone to say I understand how I feel, and everything will be okay and to just take it slow and take one step at a time and not rush or feel overwhelmed and tired.

#### Pain Management Concerns

One of the most consistent themes across most of the users was pain management. Users would mention feeling “scared” of recurrence and of “living with it for a lifetime.” They would write about the intensity of pain and the difficulty in managing pain. Many directly asked Wysa for pain relief techniques and tools, for example, “Have you got any coping skills for chronic pain?” Some would state it as a goal for themselves, mentioning that they needed “help learning to live with chronic pain.” The want to learn pain acceptance was portrayed by a user who wrote the following:

I'm trying to be more compassionate with myself, but I have not been compassionate or patient with those parts of myself and the disorder. … But if I can accept it anyway, I might have a chance at relief. ….. I am listening to my body even when it is hard and even when it's not a perfect…. glamorous process. It's usually not.

### Objective 2: App Engagement and Disengagement of Users With Chronic Pain

**Figure 2:**
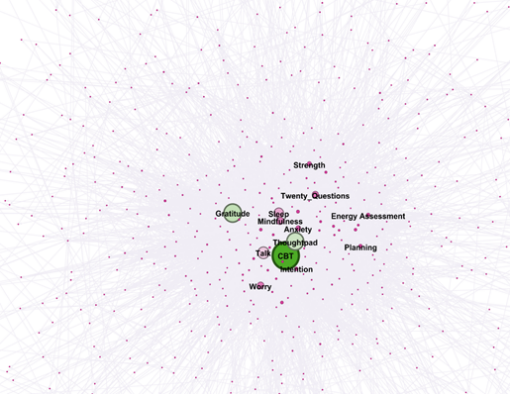
The most frequently used conversational flows for users with chronic pain (N=2194).

The frequency of the tools used indicated that across all tool usage by users with chronic pain within the defined time period in Wysa, users used gratitude (22%), sleep meditations (20%), anxiety management and modulation (10%), mindfulness meditations for self-compassion (10%), thought recording (7%), and conflict resolution exercises (3%) most frequently. The frequency of engagement within the app was further validated by examining the conversational flows through a network analysis mapped using cleaned and weighted data points, which depicted similar usage (shown in Figure 2).

To evaluate the intensity of engagement, the number of paths for users was calculated for both groups, along with the length of the interaction in each pathway. For paths of all lengths (any, minimal, and above the threshold), the difference in engagement for users managing chronic pain was found to be significant using the Mann-Whitney *U* test, with a large effect size, as reported in Table 2.

Additionally, the analysis indicated that the points of disengagement (where users stopped engaging with the intervention) were from the same set of tools where the highest engagement was also noted. Gratitude, sleep, and thought recording were the most prominent in this list.

**Table 2:** Mann-Whitney U test for the difference in app engagement between users with chronic pain and those without chronic pain.

Variable	Users with chronic pain, n	Users without chronic pain, n	Effect size (Vargha and Delaney *A*)	*U*	*P* value
Paths of any length	2194	1880	0.803 (large)	3314141	<.001
Paths below the threshold for conversational units	1737	1258	0.760 (large)	1661594	<.001
Paths above the threshold for conversational units	2185	1525	0.791 (large)	2636905	<.001

### Objective 3: Efficacy in Improving the Mental Health of Users With Chronic Pain

For both PHQ-9 and GAD-7 analyses, the paired *t* test indicated that there was a significant difference in the group means of the first and last assessments (PHQ-9: mean 13.22, SD 5.04 vs mean 10.03, SD 5.55; *P*<.001; GAD-7: mean 12.02, SD 4.47 vs mean 9.04, SD 5.15; *P*<.001) at the 95% confidence level, with medium effect sizes (PHQ-9: Cohen *d*=0.600; GAD-7: Cohen *d*=0.616).

## Discussion

This study aimed to evaluate the perceived needs of users, the engagement of users, and the effectiveness of Wysa for mental health outcomes among users who reported chronic pain and engaged with the CBT-based digital health app for support.

The thematic analysis indicated a number of mental health concerns that chronic pain users have, including emotional difficulties and self-image–related concerns. This observational study captured the experience of these difficulties through messages. The findings are consistent with the existing literature, which highlights the association of negative emotional experiences, such as anxiety and depression, with chronic pain [28,29]. Consistent with the literature, this study highlights the bidirectional relationship between pain and health (including physical health and mental health). The emerging themes from the qualitative analysis indicate that factors, such as stress, sleep disturbances, and anxiety about health, appear to influence the physical and mental health of users with chronic pain and also affect its relationship with pain. This relationship between mental health and chronic pain highlights the importance of the treatment of mental health conditions in chronic pain [30,31].

The conversational pathways frequented and the tools used reflected the needs that clients expressed to the AI conversational agent. For instance, the users expressed their distress regarding disturbed sleep, difficulty in managing negative thoughts, difficulty in interpersonal relationships, and self-image issues. Consistent with the perceived needs and the distress that users with chronic pain reported, the tools most used and conversational pathways most frequented involved techniques and tools that address these concerns, such as exercises for cognitive reframing, thought recording, gratitude, mindful compassion, sleep and mindfulness meditations, mindful compassion meditation, and conflict resolution exercises.

The user messages also depicted the isolating nature of pain, and how chronic pain users often found themselves with limited social interaction, and scarce understanding and support from family. It is thus not surprising that one of the most common conversational flows for need fulfillment was thought recording, where the users could openly write to Wysa about their thoughts and dilemmas. Being able to offer a space for disclosure is also perhaps indicative that AI apps are able to have authentic and human-level therapeutic bonds [32], which is perhaps reinforced because of the nonjudgemental atmosphere, complete anonymity, accessibility, and constant availability that the app provides.

An analysis of the in-app engagement of chronic pain users depicted a high need for support, evidenced by the number of sessions and the length of each session, which was significantly higher compared to nonchronic pain users on the app. The qualitative analysis further elaborated on the needs of the users by capturing the immense impact of chronic pain on their lives, which extended from their physical and mental health to the socioeconomic aspects of their lives. The themes revealed that the users have concerns far beyond medical services that are generally provided. Such needs have been documented in previous studies [33-35], and the findings of this study further reinforce this.

It is also important to note that while these are the most common conversational flows and most used tools, some of these app elements and tools also represent points at which some users disengaged with Wysa. This disengagement highlights that while there is a strong need, perhaps these users who disengage do not have the necessary resources or energy levels to engage with the app, given the overwhelming number of concerns that the users with chronic pain have to deal with, as indicated in the themes. Additionally, processes, such as catastrophizing and low frustration tolerance, have been found to be involved in the cognitive processes of chronic pain patients [36]. It is possible that low frustration tolerance along with an overwhelming number of concerns caused some chronic pain users to want quick relief solutions, as often evidenced in clinical practice [37], leading them to disengage with these tools due to frustration.

The findings of this study also indicate that users not only expressed the need for learning the skills of acceptance (as indicated in the theme *pain management concerns*), but also used the tools of CBT and Mindfulness, such as cognitive reframing exercises, gratitude exercises, mindful compassion, sleep meditations, and mindfulness meditations, as observed through path analyses. This perhaps indicates that users with chronic pain not only found these tools useful, but also continued to take these conversational paths multiple times because of the perceived usefulness. Our study further revealed significant improvements in both anxiety and depression in a subsample of users who completed the preassessment and postassessment. This evidence suggests that individuals with chronic pain can benefit from the CBT and acceptance-based tools present in Wysa. The value of these findings is highlighted when examined in light of prior literature where approaches, such as CBT, mindfulness, and acceptance-based interventions (eg, mindfulness-based stress reduction), have been found to be efficacious in the treatment of chronic pain [30,38,39] and are recommended lines of treatment.

This study has several limitations, and the findings should be interpreted in light of these limitations. This study is limited by the retrospective observational design. As such, the sample was nonrandomized, and this approach also precluded any conclusions of the causality of the effectiveness of interventions. The study was further limited because users were not required to complete assessments, limiting the study of efficacy to a small subsample. A major limitation of this study is that the data extraction keywords used were based on the guidelines of the International Association for the Study of Pain, extant literature, and the clinical experiences of the study researchers. However, despite efforts to have multiple sources for these keywords, these may not have included relevant terms or descriptions of pain. Another limitation is that the repeated measurements for efficacy, without a control group, could have raised the risk of regression to the mean [40]. The nonrandomized sampling of the design also limits the generalizability of the findings. Additionally, the third objective of the study was based on small samples, which further limits the generalizability of the findings of effectiveness, and the results should be interpreted as preliminary outcomes.

Despite the limitations, the findings of this study have important implications. This study, through its unique approach of user-led research, has highlighted the perceived needs and digital engagement patterns of users with chronic pain. Having a comprehensive understanding of the perceived needs and most frequented conversational paths of the users with chronic pain will help toward developing specific interventions, with improved product design and user experience. This could further help to study the effects of the interventions on a larger sample, with control groups (to account for effects such as regression to the mean), and to draw more generalizable conclusions. The study shows promising preliminary results for the use of AI in ameliorating mental health concerns among people with chronic pain. The findings suggest that digital interventions involving acceptance, CBT, and mindfulness-based therapies could be effective in meeting the needs, and could begin to bridge the gap between the demand for mental health support and the lack of adequate resources or personnel.

The findings of this retrospective study help in understanding the pervasive concerns and perceived needs of chronic pain users, and the patterns of their engagement and disengagement with the CBT-based AI mental health app Wysa. The results indicated clinically meaningful and significant improvements in the anxiety and depression scores of users with chronic pain. Though this study is limited by its retrospective design, it provides promising results for filling the gap within available treatments and supporting the needs of users with chronic pain through the use of digital mental health interventions.

## Abbreviations

AI: artificial intelligence
CBT: cognitive behavioral therapy
GAD-7: Generalized Anxiety Disorder Assessment-7
PHQ-9: Patient Health Questionnaire-9
